# Predicting Vasovagal Reactions to Needles from Facial Action Units

**DOI:** 10.3390/jcm12041644

**Published:** 2023-02-18

**Authors:** Judita Rudokaite, Itir Onal Ertugrul, Sharon Ong, Mart P. Janssen, Elisabeth Huis in ‘t Veld

**Affiliations:** 1 Department of Cognitive Science and Artificial Intelligence, Tilburg University, Warandelaan 2, 5037 AB Tilburg, The Netherlands; 2 Donor Studies, Department of Donor Medicine Research, Sanquin Research, Plesmanlaan 125, 1066 CX Amsterdam, The Netherlands; 3 Department of Information and Computing Sciences, Utrecht University, Heidelberglaan 8, 3584 CS Utrecht, The Netherlands

**Keywords:** vasovagal reactions, needle fear, machine learning, facial action units, blood donors

## Abstract

Background: Merely the sight of needles can cause extreme emotional and physical (vasovagal) reactions (VVRs). However, needle fear and VVRs are not easy to measure nor prevent as they are automatic and difficult to self-report. This study aims to investigate whether a blood donors’ unconscious facial microexpressions in the waiting room, prior to actual blood donation, can be used to predict who will experience a VVR later, during the donation. Methods: The presence and intensity of 17 facial action units were extracted from video recordings of 227 blood donors and were used to classify low and high VVR levels using machine-learning algorithms. We included three groups of blood donors as follows: (1) a control group, who had never experienced a VVR in the past (*n* = 81); (2) a ‘sensitive’ group, who experienced a VVR at their last donation (*n* = 51); and (3) new donors, who are at increased risk of experiencing a VVR (*n* = 95). Results: The model performed very well, with an F1 (=the weighted average of precision and recall) score of 0.82. The most predictive feature was the intensity of facial action units in the eye regions. Conclusions: To our knowledge, this study is the first to demonstrate that it is possible to predict who will experience a vasovagal response during blood donation through facial microexpression analyses prior to donation.

## 1. Introduction

The journey of a bag of blood, which ends up potentially saving the life of a patient through a blood transfusion, invariably starts with the willingness and ability of a person to donate blood. Unfortunately, needle-related procedures, such as blood donations, have the unique ability to cause adverse emotional and physical reactions (so-called vasovagal reactions (VVRs) in the donor, including, but not limited to, nausea, dizziness, sweating, pallor changes, or even severe symptoms, such as fainting with a loss of consciousness [1,2,3]. Even though the majority of VVRs do not have many long-lasting physical effects on blood donors [4,5], they are very unpleasant to experience, and increased anxiety and needle fear is sufficient to negatively impact donor return [6,7,8]. The risk of VVR is closely related to the experience of negative emotions, such as fear, anxiety, and stress [6,7,8,9,10,11,12], and to physiological changes such as increased heart rate [13] or changes in breathing patterns [14], controlled by the sympathetic autonomic nervous system (ANS). Previous research has shown that these physiological responses are already happening prior to the blood donation, peaking at the moment the needle is inserted [15,16]. Research has also shown that these processes are very difficult to self-report until it is too late. Indeed, in their review on risk factors for VVRs, Thijsen and Masser (2019; [3]) classify risk factors such as heartrate and (anticipated) anxiety, fear, and pain as ‘unobservable donor characteristics’ and indeed, most previous studies have mostly relied on subjective self-reporting through questionnaires, etc. [17,18,19,20]. However, currently used interventions to prevent VVRs in the blood donation setting, such as applied muscle tension (AMT) or water loading, aim to reduce the symptoms related to the loss of a pint of blood [9]. These interventions are proven to be effective [21] but may therefore not work for donors who suffer from VVRs due to negative, anticipatory emotions [10,11,12], and not all donors or blood collection staff are happy with AMT [9,22,23]. Furthermore, AMT, as well as monitoring and social support from the blood donation staff, takes place when the donor is already in the donation chair, but anticipatory anxiety already plays a role when the donor is in the waiting room [15,16], where they are usually unsupervised by the blood collection center staff. There is clear evidence that targeting anticipatory worries, anxiety, or negative emotions in an early stage may be a promising avenue for further decreasing the level of VVRs [23].

Hence, to address some of the problems with current interventions, as well as some additional non-addressed needs, we are developing a serious game that the donor can play unsupervised prior to the donation that targets the ‘unobservable donor characteristics’, which can be a valuable addition to other interventions, such as water loading and AMT, and hopefully reduces the prevalence of VVRs during the donation, thereby decreasing the burden on blood collection center staff to intervene during the donation. 

The aim of the AINAR game is to make the unobservable observable, and to help the donor learn how to control their anticipatory symptoms of VVR and anxiety using biofeedback. The following question then arises: what is the most promising method of measuring these subtle anticipatory processes in the face that can be performed by a smartphone? In this study, the effectiveness of the automatic extraction of facial muscle movements from video, using the Facial Action Coding System (FACS) [24], is assessed. The FACS is the most comprehensive, anatomically based system for describing facial movements [24]. It consists of action units (AUs) related to the actions or movement of (groups of) muscles in the face. Previous studies show that the FACS was successfully used for detecting anxiety, stress, fear (e.g., [25,26]), and pain (e.g., [27,28,29]). For example, stress is associated with raised cheeks, jaw drop, pulled lip corners, and tightened eye lids [30] whereas typical AUs associated with pain expression include brow lowering, cheek raising, lid tightening, upper lip raising, and eye closing [31,32,33]. Fear can also be detected from minimal cues in the upper face, specifically by eyebrow and eyelid raising and brow furrowing [33]. Anxiety can be detected from a combination of both the upper and lower face, including a raised outer brow, wrinkled nose, and parted lips [25]. As emotions such as pain, anxiety, stress, and fear are also observed during injections [34], it is expected that subtle changes in muscle movements could be measured during the anticipatory waiting time prior to the donation and used to predict VVRs during the donation.

The aim of this study is to develop a machine-learning method that is able to monitor donors’ well-being by detecting automatic, minute changes in their facial muscles through video analyses. Even more specifically, we aim to predict if a donor will suffer from overt adverse emotional or physical responses during the donation by monitoring their covert, automatic facial reactions in the waiting room.

## 2. Materials and Methods

### 2.1. Donor Recruitment

Participants were recruited from the regular blood donor pool from Sanquin, the not-for-profit organization responsible for blood and plasma collection, processing, and distribution in the Netherlands. The study took place at three blood collection centers (BCC; Leiden, ‘s-Hertogenbosch, and Zwolle). All blood donors who fit into the following three groups were invited to participate: (1) a control group with between 5 and 10 previous donations but no previous experience of vasovagal reactions; (2) the sensitive group with between 5 and 10 donations but who experienced a VVR at the previous donation; and (3) a new donor group, consisting of first-time donors. The study was approved by the Ethics Advisory Board of Sanquin. All donors provided informed consent.

### 2.2. Procedure

Interested donors contacted the data manager for an appointment and received information about the study, including ethical consent information.

On arrival, participants completed a questionnaire containing items regarding needle fear and various personality questionnaires (T = 20–25 min). Directly after, a one-minute video was recorded and the first VVR measurement was taken (stage 1). Next, the donors proceeded with the regular blood donation procedure, containing six more stages during which the participants were recorded and VVR measurements were taken, starting after the donors completed the standard registration form at Sanquin (stage 2). Then, the donors were sent to consult the physician who checked the donor for donation eligibility. If the donor was deferred for donation (for example, due to having low hemoglobin levels) their participation stopped. Then, depending on the BCC location, which all have slightly different ‘donor routes’, the donors either had to wait to be called for donation in the waiting area, or they were directly sent to the donation chair. If the donor had to wait in the waiting area, we again asked them to self-report VVR and recorded a third video, lasting around 1 to 2 min (stage 3). If the donor was sent directly to the donation chair, we proceeded with a continuous video recording in the donation chair (stage 4–6), lasting around 5–27 min. During the video recording, we accessed VVR levels three times as follows: at the needle insertion moment (stage 4); at around 300 mL of donated blood (stage 5); and during needle uncoupling (stage 6). The final recording and VVR level assessment took place in the waiting area, where the donors could recuperate from the donation (stage 7). The donors were free to behave as they normally would throughout the procedure. The VVR ratings were verbally taken by the data manager, and the respondents also answered verbally.

### 2.3. Materials and Measures

Video recording. The video was recorded at 25 frames per second using the Nikon Coolpix AW130. The camera was installed on a tripod at a distance of about 1 m from the donor. 

Vasovagal reactions (VVR levels). At each of the seven stages, participants were asked to rate on a Likert scale from 1 (not at all) to 5 (extremely) to what extent they experienced faintness, dizziness, weakness, and lightheadedness (partly based on the Blood Donation Reactions Inventory [35]). Furthermore, they were asked to rate emotional responses, i.e., fear, stress, tension, and nervousness, using the same scale.

The ratings of stages 1 and 2 were used as predictors (features) in the models. The ratings of the last four stages (4–7) were summed, resulting in a score between 32 and 160, which served as the dependent variable.

### 2.4. Video Data Preprocessing

The video data from stages 1 and 2 were combined into a continuous time series (N = 2000 frames). Then, the presence and intensity of 17 action units at each frame were extracted using OpenFace [36]. AUs’ presence indicated whether the AU was visible in the face or not (0 = not present, 1 = present), and AU intensity showed how intense the AU (minimal to maximal) was on a 5-point scale, with 5 being the highest value. The following seventeen AUs were assessed: AU1 (raised inner brow); AU2 (raised outer brow); AU4 (lowered brow); AU5 (raised upper lid); AU6 (raised cheeks); AU7 (tightened eye lids); AU9 (wrinkled nose); AU10 (raised upper lip); AU12 (pulled lip corner); AU14 (dimples formed); AU15 (lowered lip corners); AU17 (raised chin); AU20 (stretched lips); AU23 (tightened lips); AU25 (lips apart); AU26 (jaw drop); and AU45 (blink) [36]. 

We followed two approaches to extract the features of AUs for machine-learning models and compared the performance of both datasets:

1. Presence. The presence of the 17 AUs was calculated as the total sum of present AUs per frame, divided by the total number of frames, resulting in the proportion of AU presence per participant ranging from 0 (never active) to 1 (always active) (total number of extracted features = 17).

2. Intensity. Using the Tsfresh python package [37], six intensity level characteristics of all seventeen AUs were automatically extracted, namely the sum, variance, standard deviation, maximum, mean, and mean-root-square values (total number of extracted features = 102). 

### 2.5. Model Training, Validation, and Evaluation

Each data set was split into a train (80%) and test (20%) set, on which the model performance was assessed. The data were scaled using a standardization technique where the values centered around the mean with a unit standard deviation. 

Because the donor dataset was highly positively skewed (*n* = 163 in with a low VVR level versus *n* = 64 with a high VVR level) and standard machine-learning techniques tend to have a bias towards the majority class if there is an imbalance, the synthetic minority oversampling technique (SMOTE) [38] was applied to tackle this issue. Specifically, SMOTE synthesizes new instances of the minority class (high VVR group) by randomly selecting one or more examples of the minority class and then determining the vector between that data point and the chosen k-nearest neighbors in order to randomly generate a new example. The created synthetic data points are then added to the training set to match the size of the majority class (low VVR group) that equal the size of the low and high VVR examples be present in the training set.

In a machine-learning study like this, there are many predictor variables, which makes it imperative that the model is trained well on the training data without introducing bias. Therefore, while training the model, a resampling procedure called nested k-fold cross-validation was used to evaluate the machine-learning models on a limited data sample [39]. To prevent the overfitting of the model and to prevent an overly optimistic evaluation, we applied a common method for predictor (feature) selection called recursive feature elimination with cross-validation (RFECV; [40]) and hyperparameter tuning using GridSearchCV [41]. This is a way of pruning predictors until an optimal set is reached (for more information and an overview of the hyperparameters explored for each algorithm, see Appendix A, Table A1) [41]. Furthermore, how well the classification algorithms performed was assessed. Due to class imbalance and our interest in correctly classifying the minority group (blood donors who are at higher risk of experiencing VVR), we decided to evaluate our model performance on the following metrics:

1. Precision. Precision is the proportion of true results over all the examples that were predicted to belong to a certain class. In this study, precision ensured that we did not misclassify too many donors as experiencing VVR when they did not experience it. 

2. Recall. Recall is the fraction of examples that were predicted to belong to a class with respect to all the examples that truly belong in the class. In this study, a high recall value ensured that blood donors who experienced VVR were not overlooked. 

3. F1 score. The F1 score is the harmonic mean of precision and recall. The higher the score, the better the model’s performance.

4. AUC-PR score. The area under the precision–recall curve summarizes a precision–recall curve as the weighted mean of precision values over all recall values. The higher the score, the better the performance of the model, with 1.0 representing a perfect model.

**Figure 1:**
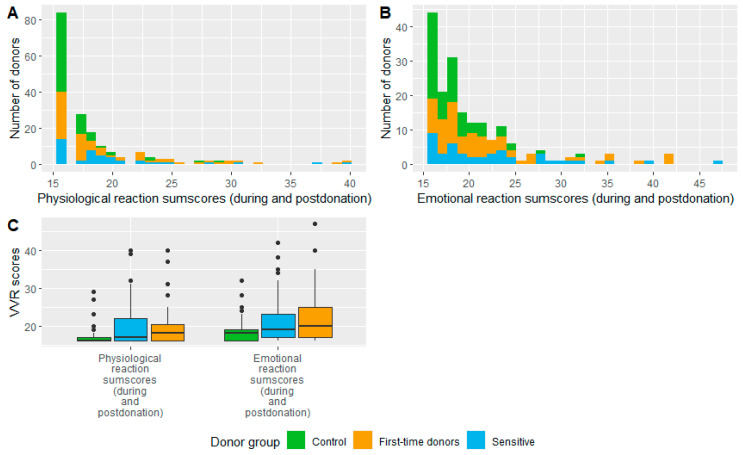
The figure represents distribution (**A**,**B**) and the mean and variation (**C**) in total physiological (**A**) and psychological (**B**) scores that were reported during and post-donation (specifically, during stages 4–7) per group. The line in the box (**C**) represents the mean of each group and the dots above the box represent the outliers per group.

The model was trained using four machine-learning algorithms: a decision tree; a random forest classifier; an XGBoost; and an artificial neural network. As the baseline model, the self-reported pre-donation VVR scores from stage 1 and stage 2 were used as model inputs (see Figure 1 for the score distribution).

To build, tune, and evaluate the models, Scikit-Learn [42], XGboost [43], Tensorflow [44], and Keras [45] in Python were used. The Matplotlib library in Python [46] and ggplot2 [47] in RStudio [48] were used for visualization. The SHAP (SHapley Additive exPlanations) package in Python [49] was used to explain the output of the best performing machine-learning model. SHAP values interpret the impact of particular values for a given feature relative to the prediction for which that feature was at baseline.

## 3. Results

### 3.1. Participants

Data was collected from *n* = 227 blood donors in total (control group: *n* = 81; sensitive group: *n* = 51; new donors: *n* = 95). No significant gender differences (*F(2)* = 1.56, *p* = 0.2) or BCC location differences (*F(2)* = 1.36, *p* = 0.3) were found between the groups.

### 3.2. Physical and Emotional Reactions

We first separately analyzed physiological and psychological VVR levels to evaluate whether there were any significant differences between the subscales before summing them as a total score. One-way ANOVA showed a statistically significant main effect on the group of the total physiological VVR levels during stages 4–7 (*F*(2) = 7.658, *p* < 0.001). The control group experienced significantly lower VVR levels than the sensitive (*p* < 0.006) and new donors (*p* = 0.002) groups, but no significant difference was found between the sensitive and new donor groups (*p* = 0.999; Figure 1C). 

The same pattern was found using one-way ANOVA with the total score of negative emotional ratings during stage 4–7 (*F*(2) = 8.499, *p* < 0.001) where the control group experienced significantly lower levels of adverse emotions than the sensitive (*p* < 0.001) and new donors (*p* < 0.002) groups, but no significant differences were found between sensitive and new donor groups (*p* = 0.81, Figure 1C). Figure 1A,B show the distribution of the sum scores on both physiological and emotional reaction levels. As the pattern of the results are very similar, we combined the scores together and analyzed the results further using an overall score, referred to as the VVR score.

### 3.3. Overall VVR Scores

**Figure 2:**
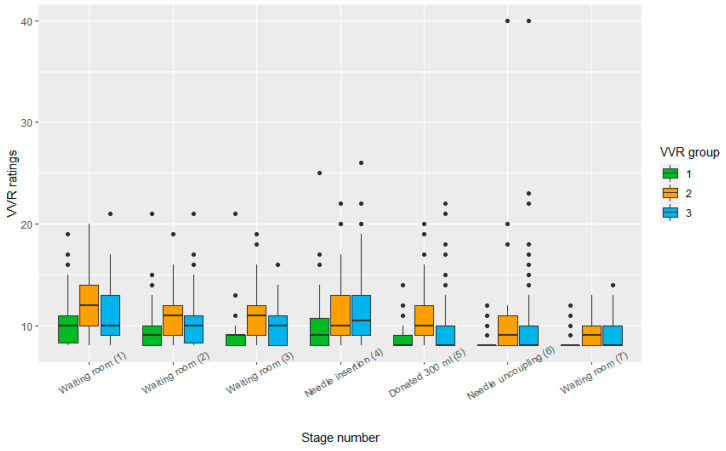
Distribution of VVR ratings per stage and group. The dots above the box represent the outliers per group.

The VVR scores were positively skewed, reflecting a high proportion of blood donors who reported low VVR scores (M = 39.55, SD = 9.75, median = 32; min = 32, max = 81, see Figure 2). Six participants (3%) experienced severe symptoms of vasovagal syncope (fainting), which resulted in interference by a donor assistant. 

**Figure 3:**
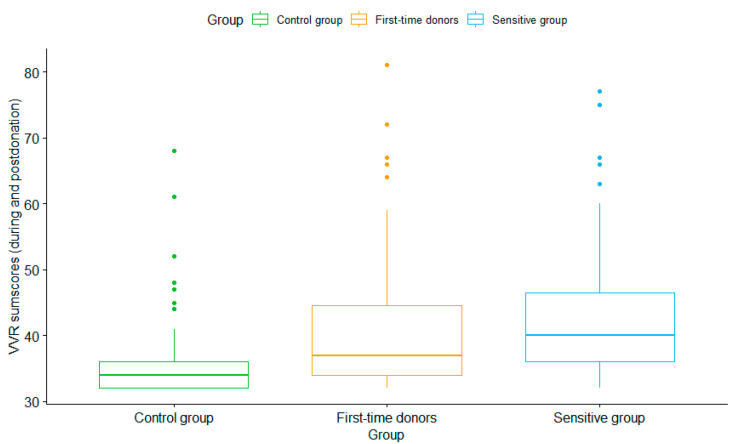
Variation in total VVR scores (during stages 4–7) per group. VVR symptoms consist of faintness, dizziness, weakness and lightheadedness, fear, stress, tension, and nervousness. The line in the box represents the mean of each group and the dots above the box represent the outliers per group.

One-way ANOVA showed a statistically significant main effect on the groups of the total VVR symptoms during stages 4–7 (*F*(2) = 11.66, *p* < 0.001). The control group experienced significantly lower VVR levels than the sensitive (*p* <= 0.0001) and new donor (*p* = 0.003) groups, but no significant differences were found between the sensitive and new donor groups (*p* = 0.16; see Figure 3). 

**Figure 4:**
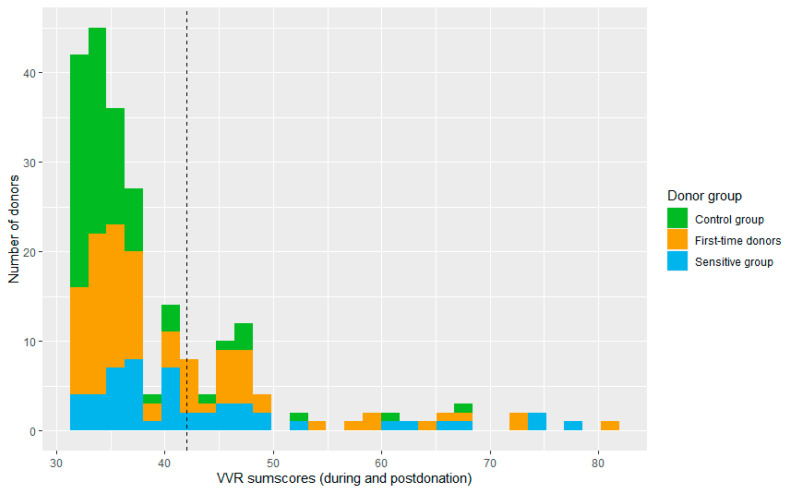
Distribution of total VVR scores during stages 4–7. The black dashed line represents the mean-based cut-off of the sample based on which the low vs high VVR groups were split.

The sample was split based on the mean into a low VVR score group (*n* = 163, VVR score ≤ 41) and a high VVR score group (*n* = 64, VVR level > 41, see Figure 4). 

### 3.4. VVR Classification Results

Four machine-learning algorithms were applied to the extracted AU characteristics in order to classify which donors would experience low or high levels of VVR during the donation. As the baseline model, only the self-reported pre-donation VVR scores were entered (N = 2, stage 1 and stage 2). Next, the performances of various machine-learning algorithms on the extracted action unit characteristics (presence and intensity with RFECV) on the test set were compared (see Table 1). The performance of all machine-learning algorithms using AU presence and intensity without RFECV showed slightly lower performance. 

**Figure 5:**
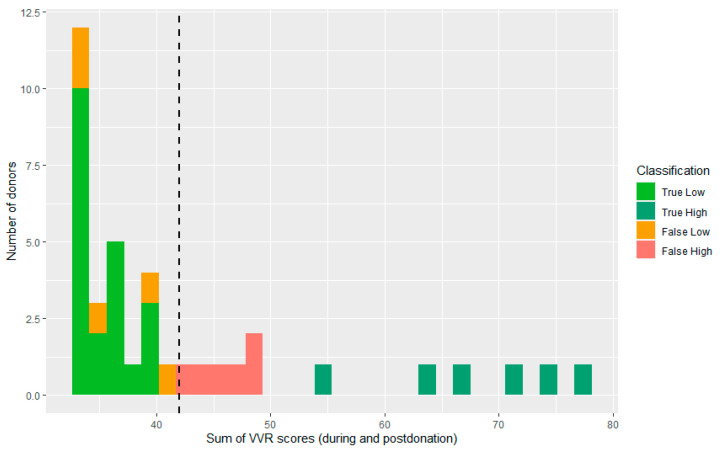
Figure shows correctly (dark and light green) and incorrectly (orange and red) classified samples on the test set using neural network.

Models that only included the self-reported pre-donation VVR scores already showed a good performance, with F1 scores varying from 0.69 to 0.77. However, a much better predictive performance (F1 score = 0.82) was achieved by looking at the facial action unit intensities using a neural network algorithm (see Table 1). Figure 5 shows where the model made errors in the classification, which shows that this model was able to correctly classify all donors who reported high VVR levels on the test set (see yellow bars in Figure 4). The performance was less good for donors whose VVR levels were around the cut-off point. As can be seen by the green bars in Figure 5, some donors scored around the midrange in terms of vasovagal reactions (between 42 and 50 points).

**Figure 6:**
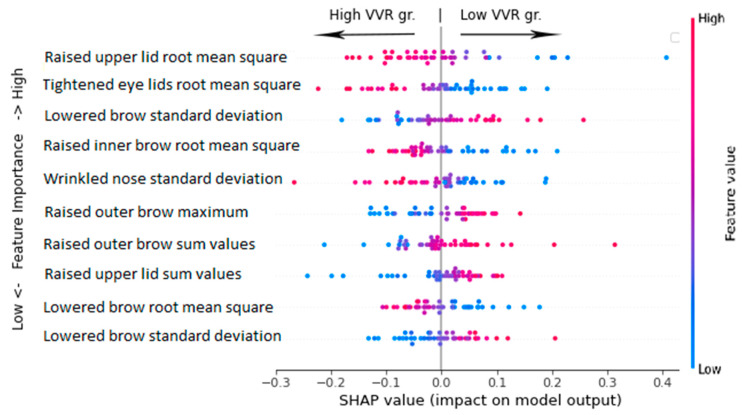
The neural network performance evaluation on the test set using intensity of AUs with RFECV (N features = 41). The performance evaluation on the test set using neural network classifier (N features = 41, achieved F1 = 0.82). The figure represents the feature impact on the model output based on the game theoretic approach SHAP (SHapley Additive exPlanations). The SHAP summary plot combines feature importance (*y*-axis) with feature effect (*x*-axis), where each point represents a SHAP value. All features are sorted by importance from the highest to the lowest. Blue color indicates low values and red color indicates high values of the given facial action feature. The negative score on the *x*-axis is associated with the ‘high VVR’ group and a positive score on the x-axis is associated with the ‘low VVR’ group. For example, the higher the root mean square of the movements of the upper eyelid (‘AU05’), the higher the chance that the blood donor is classified in the high VVR group. Similarly, the lower the root mean square of brow movements (‘AU07’), the higher the chance the blood donor will be classified as being in the low VVR group.

The most important predictors in the best performing model were those located in the mid and upper regions of the face, including movement in the eyelids (raising the upper lid, tightening the eyes), movement in the eyebrows (related to moving the brows up and down), and wrinkling the nose (Figure 6). For example, the most important feature of the ‘root mean square’ of the intensity of a raised upper lid can be interpreted such that the strength of upper eyelid intensity was the most important feature in differentiating between low and high VVR groups. In simple terms, the more raised and tightened the upper eyelid was over time, the more likely the model was to classify a person as being in a high-risk VVR group.

**Figure 7:**
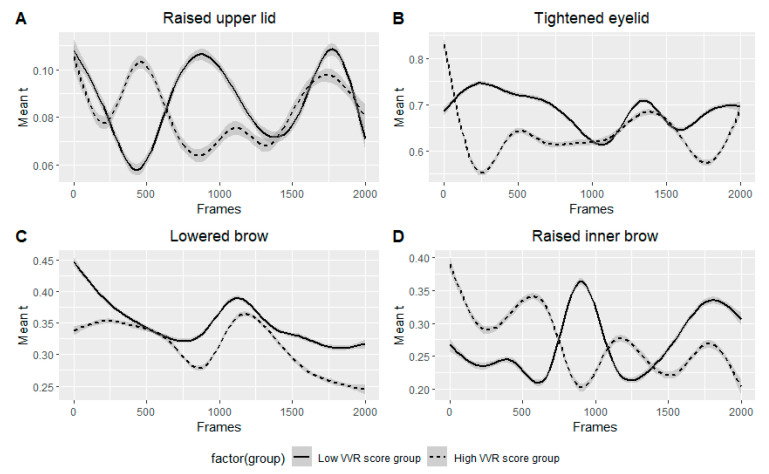
A smoothed visual representation of the mean intensity of the four most predictive action units between low and high VVR groups during the 2000 frames prior to the donation. (**A**) shows the mean intensity value of raised upper eyelid, (**B**) shows the mean intensity value of tightened eyelid, (**C**) shows the mean intensity value of lowered eyebrow, and (**D**) shows the mean intensity value of raised inner eyebrow.

Then, the standard deviation of specific action unit represents a deviation from the mean of the specific AU intensity and the maximum value represents the highest reached intensity of the specific action unit. For instance, the higher the deviation from the mean of a lowered brow and the higher overall intensity of a raised outer brow, the more likely it is that the donor belongs to the low VVR group. See Figure 7 for the average intensity of the four most important predictors per risk group.

## 4. Discussion

In this study, we measured whether emotional microexpressions, measured as activity in the facial muscles prior to blood donation, can indicate whether a donor will experience a vasovagal reaction during blood donation. Firstly, the results show that first-time donors and donors who experienced a VVR before reported higher levels of physical and emotional reactions during donation than the control group, consisting of experienced donors who had never before experienced a VVR. This corroborates previous findings that anxiety, pain, and fear of needles are risk factors for experiencing VVRs during the donation, especially in new donors and donors with a history of VVRs [50,51]. Secondly, we ruled out that the experience of a VVR during the donation could be predicted only by the subjective (self-reported) VVR ratings of the donor prior to the donation, without taking action unit activity into account. Even though these models performed fairly well (with an F1 score of 0.77), the precision of the models with only the pre-donation self-reported ratings were higher than their recall, indicating that these models did not manage to correctly identify the donors who experienced high levels of VVR very well. 

On the other hand, the performance of the models that only included the intensity of facial muscle activation that occurred in the waiting room resulted in a better performance, with a higher F1 score of 0.82. This demonstrates that subtle, unconscious, and automatic facial muscle movements are a better target for predicting adverse emotional or vasovagal reactions during the donation than self-reports. Importantly, this model was also the best at identifying donors who experienced high levels of VVR, with a recall of 0.84. This is in line with previous research showing that bodily responses measured objectively (such as heart rate, blood pressure, and cortisol) are present before donors report overt symptoms of VVRs [15,16].

**Figure 8:**
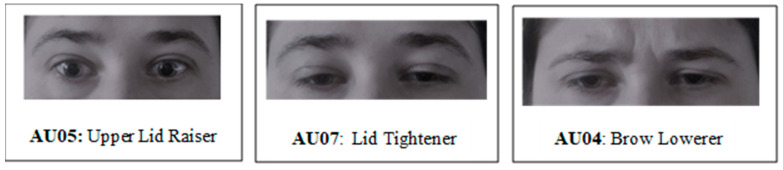
The most discriminative facial regions found in neural network model (based on Facial Action Coding System; [24]).

When it comes to predicting who will experience a VVR during the donation, the most discriminative facial regions found in the best performing neural network model were found in the upper part of the face. More specifically, stronger movements in the eye region, such as raising the upper eyelids (AU05), as well as tightening the eye lids (AU07) and raising the inner brow (AU01), indicated high risk for VVR. Additionally, a stronger but more constant activity in the action unit responsible was ‘lowering the brow’ (AU04) or in other words, donors who are squinting more strongly and consistently are also at higher risk for VVR. On the other hand, donors who showed more variation in this ‘squinting’ behavior (as shown by higher standard deviations in the intensity of this action unit) were more likely to not experience a VVR during the donation (see Figure 8 for the most important AU).

Overall, these features are often associated with fear and pain responses [52,53], and AU intensity is significantly increased during the stress response, resulting in much more expressive human face expressions [33].

A limitation of this study is the decision on how to divide donors into a low versus high VVR group. In this study, it was decided to use a mean split of the total level of emotional and physical reactions, and most of the misclassifications occurred around this cut-off. This is not unexpected, but it is still a drawback of this study. A mean split was chosen not only because only a few donors reported extremely high VVR levels, but also because we aimed to develop a method which could also work for those donors who have subtler experiences of VVR than full-fledged fainting. That said, it is expected that the levels of experienced fear and vasovagal reactions would be lower in a sample of blood donors as opposed to, e.g., a sample of patients in a hospital. In general, the VVR scores found in our study are comparable to the previously reported prevalence of very overt VVR reactions during blood donation (ranging from 0.1% to 0.5%, [2]). Currently, the research team is collecting more data, especially from new and ‘sensitive’ donors (who previously experienced vasovagal reactions), as they tend to show higher levels of self-reported VVR, which in the future may improve the performance of the models. Additionally, we are developing similar methods testing other features, such as facial thermal patterns (submitted), automated heart rate, and respiration rate extraction (in preparation), as well as a similar method that uses the video stream to directly classify high and low VVR groups without extracting specific features using Long Short Term Memory networks (LSTM) or Gated recurrent units (GRU) models (submitted) and to model the outcome without a cut-off level. 

As mentioned before, the aim of this line of research is to develop a solution that is able to not only identify those at risk for VVR prior to a needle-related procedure using a donor’s own smartphone, but that is simultaneously able to teach donors or patients how to control and prevent VVRs from happening at all. This has resulted in a prototype of our AI-driven biofeedback game, AINAR.io. The aim of algorithms such as the one described in this paper is to implement donors into the AINAR game where they control the biofeedback loop. In the game, you play an innocent, unrelated slide and fly game. The AINAR game monitors the face of the player through the front-facing camera and, using the algorithms we are developing, gives the player continuous feedback on their well-being in a subtle and playful way. When the donor is doing well, the weather in the game is sunny. However, if the algorithms pick up risk factors which signal the donor is at risk for experiencing fear or a VVR, it may start to rain or even start to snow. The player at that point probably still feels fine, which is also visible in the self-reported ratings of emotional and physical reactions, which are low prior to the donation. However, the changing weather is a signal to the player that they should start to see how they can control and prevent their emotional and physical reactions. The game will not tell them how to perform this directly. Instead, the player should try to experiment with strategies, which can include, but are not limited to, paying attention to breathing, relaxation or mindfulness, thinking about something else, or anything else that the donor themselves may expect to work. When the player uses a strategy that indeed reduces (the risk of) emotional or physical reactions, the game will immediately ‘see’ this and change the weather back to normal. This type of positive reinforcement, or biofeedback, has been shown to be effective in teaching people how to control a plethora of automatic physiological processes (e.g., heart rate, brain activity), benefiting emotional well-being in many situations and patient groups [54,55,56]. The efficacy of the AINAR game in decreasing fear and VVR will soon be tested with blood collection centers, phlebotomists, hospitals, and dentists. 

## 5. Conclusions

In conclusion, machine-learning techniques can help identify blood donors who are at high and low risk of experiencing a VVR during blood donation from the intensity of their automatic facial microexpressions around the eyes, eyebrows, and nose measured prior to the blood donation.

## Figures and Tables

**Table 1:** Machine-learning performance values on the testing set for binary classification (high/low VVR scores) with and without feature selection.

Model	Dataset Used (N = Number of Features Considered)	Performance on the Test Set			
		Precision	Recall	F1	AUC-PR
Decision tree	Pre-donation VVR ratings (N = 2)	0.85	0.68	0.75	0.88
	Intensity of AU with RFECV (N = 89)	0.71	0.78	0.75	0.79
	Presence of AU with RFECV (N = 17)	0.74	0.74	0.74	0.75
Random Forest	Pre-donation VVR ratings (N = 2)	0.86	0.71	0.77	0.87
	Intensity of AU with RFECV (N = 58)	0.72	0.81	0.76	0.72
	Presence of AU with RFECV (N = 14)	0.76	0.76	0.76	0.81
XGboost	Pre-donation VVR ratings (N = 2)	0.85	0.68	0.75	0.90
	Intensity of AU with RFECV (N = 71)	0.71	0.69	0.70	0.73
	Presence of AU with RFECV (N = 14)	0.81	0.65	0.72	0.76
Neural network	Pre-donation VVR ratings (N = 2)	0.83	0.59	0.69	0.85
	Intensity of AU with RFECV (N = 41)	0.79	0.84	0.82	0.79
	Presence of AU with RFECV (N = 8)	0.72	0.62	0.67	0.83

## Data Availability

The dataset collected during the current study is not publicly available due to participants’ privacy, but an anonymized preprocessed dataset can be requested when the FAINT study is completed by contacting the data manager of the Donor Medicine Research department at Sanquin. For contact details, contact the corresponding author or Dr. Elisabeth Huis in ‘t Veld.

## Appendix A

**Table A1:** Hyperparameter tuning using GridSearchCV for each machine-learning algorithm.

ML Algorithm	Parameter	Values/Range
XGBoost	Learning rate	[0.0001, 0.001]
	Max depth	[1, 2, 3, 4]
	Subsample	[0.6, 0.8]
	Col sample by tree	[0.5, 0.75, 1]
Decision Tree	Max depth	[2, 3, 5, 10, 20]
	Min samples leaf	[5, 10, 20, 50, 100]
	Criterion	[‘gini’, ‘entropy’]
Random Forest	Max depth	[2, 3, 5, 10, 20]
	Min samples split	[0.1, 0.3, 1]
	Min samples leaf	[1, 3, 10]
	Criterion	[‘gini’, ‘entropy’]
Neural networks	Batch size	[32, 64]
	Number of epochs	[50, 100, 200]
